# Hyperechoic breast lesions: anatomopathological correlation and differential sonographic diagnosis*

**DOI:** 10.1590/0100-3984.2014.0032

**Published:** 2016

**Authors:** Marcelo Menezes Medeiros, Luciana Graziano, Juliana Alves de Souza, Camila Souza Guatelli, Miriam Rosalina B. Poli, Rafael Yoshitake

**Affiliations:** 1 MD, Resident in Radiology, Department of Radiology and Imaging Diagnosis - A.C.Camargo Cancer Center, São Paulo, SP, Brazil.; 2 Physicians, Department of Radiology and Imaging Diagnosis - A.C.Camargo Cancer Center, São Paulo, SP, Brazil.

**Keywords:** Hyperechoic breast lesions, Ultrasonography, Breast neoplasms, Differential diagnosis

## Abstract

Hyperechoic lesions are not a frequent finding at breasts ultrasonography, and most of times are associated with benign pathologies that do not require further evaluation. However, some neoplasms such as invasive breast carcinomas and metastases may present with hyperechogenicity. Thus, the knowledge about differential diagnoses and identification of signs of lesion aggressiveness are of great relevance to avoid unnecessary procedures or underdiagnosis, and to support the correct clinical/surgical approach. On the basis of such concepts, the present essay describes and illustrates the main features of hyperechoic lesions at breast ultrasonography in different cases, with anatomopathological correlation.

## Abstract (pt)

As lesões hiperecogênicas constituem um achado com baixa prevalência nos exames ultrassonográficos das mamas, em sua maior parte associado a doenças benignas que não necessitam de avaliação adicional. Porém, algumas neoplasias, como o carcinoma invasivo da mama e as metástases, podem apresentar-se desta forma. Assim, o conhecimento dos diagnósticos diferenciais e a identificação dos sinais de agressividade das lesões são de grande relevância, a fim de evitar procedimentos desnecessários ou o subdiagnóstico, e apoiar a conduta clínica/cirúrgica correta. Com base nestes conceitos, este artigo descreve e ilustra os principais aspectos das lesões hiperecogênicas presentes no exame ultrassonográfico das mamas, por meio de diferentes casos, e sua correlação anatomopatológica.

## INTRODUCTION

Hyperechoic breast lesions are uncommon findings^(1)^ , corresponding to 5.6% of alterations identified at ultrasonography (US), with high predictive value for benignity. Such lesions correspond to 0.6% of all biopsied lesions and only 0.4% of all malignant lesions^(2)^.

Breast nodules with fatty or fibrous contents, either of vascular origin or with high cellularity may present increased echogenicity at US (Table 1). The knowledge of the characteristics of the main hyperechoic lesions, as well as the recognition of characteristics suggestive of malignancy to avoid late diagnosis might avoid many unnecessary invasive procedures^(3)^. In most cases, hyperechoic lesions are detected for being palpable or for presenting suspicious findings at mammography or magnetic resonance imaging (MRI)^(2,4)^.

**Table 1:** Types of hyperechogenic breast lesions at ultrasonography

Cause of the hyperechogenicity	Benign lesions	Malignant lesions
Lesions with fatty contents	Hamartoma Lipoma Angiolipoma Steatonecrosis	Liposarcoma
Lesions with fibrous contents	Hamartoma	Invasive ductal carcinoma
	Focal fibrosis	Invasive lobular carcinoma
	Pseudoangiomatous stromal hyperplasia	
Vascular lesions	Angiomyolipoma Hemangioma	Angiosarcoma
High-cellularity lesions	Flogosis (rare)	Invasive ductal carcinoma and ductal carcinoma in situ
	Infection (rare)	Carcinoma lobular invasivo
		Lymphoma
		Metastasis

Table adapted from Linda et al.^(1)^

The analysis of sonographic characteristics has shown that non parallel orientation and non circumscribed margins are more frequently found in malignant hyperechoic nodules than in benign ones. Such results suggest that the same sonographic characteristics utilized to evaluate hypo- or isoechoic nodules (such as spiculated margins, association with microcalcifications) should be applied in cases of hyperechoic nodules to differentiate between malignant and benign lesions^(4,5)^. Additionally, the presence of focal hypoechoic areas within hyperechoic findings increases the risk of malignancy^(2)^.

In the present study, the authors describe cases of hyperechoic breast lesions observed at US, with emphasis on the relevance of possible differential diagnoses for the correct clinical approach.

## BENIGN LESIONS

### Adenosis

**Figure 1:**
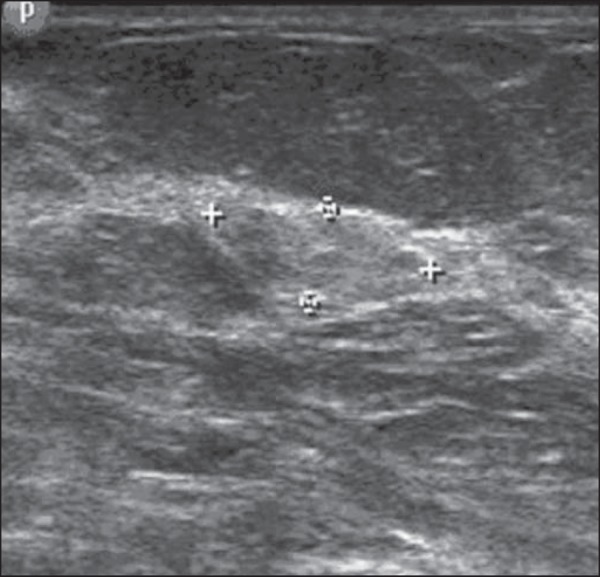
*Simple adenosis*. Ultrasonography showing hyperechoic, ovoid nodule with circumscribed margins and largest axis parallel to the skin.

Adenosis represents a wide spectrum of benign alterations of the breast tissue. In simple adenosis, there is a major preservation of the breast architecture, despite the presence of histological alterations. At US, hyperechoic areas with little or no architectural distortion are observed, since there is no stromal fibrosis (Figure 1). The sclerosing form may present architectural distortion and be associated with proliferative lesions such as intraductal papilloma, fibroadenomas, and coexist with invasive carcinomas in situ^(6,7)^.

### Hamartomas

**Figure 2:**
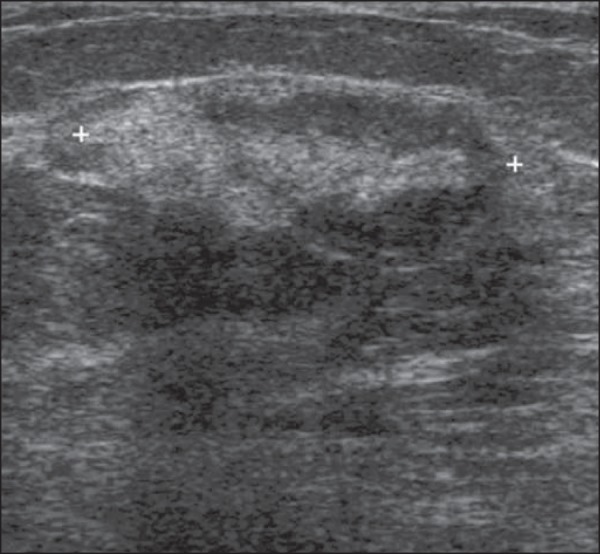
*Hamartoma*. Ultrasonography showing heterogeneous, ovoid, predominantly hyperechoic nodule with circumscribed margins, largest axis parallel to the skin, and subtle posterior acoustic shadowing.

Hamartomas are constituted of glandular, fatty and fibrous tissues, with estimated incidence of 0.1-0.7%. In most cases, they present as a mobile, barely painful nodule in middle-aged women. At US, they are nodules with circumscribed margins, peripheral halo and compressible by the transducer (Figure 2). They may be hyperechoic in 12-43% of cases, or even heterogeneous, hypoechoic and isoechoic^(8)^.

### Steatonecrosis

**Figure 3:**
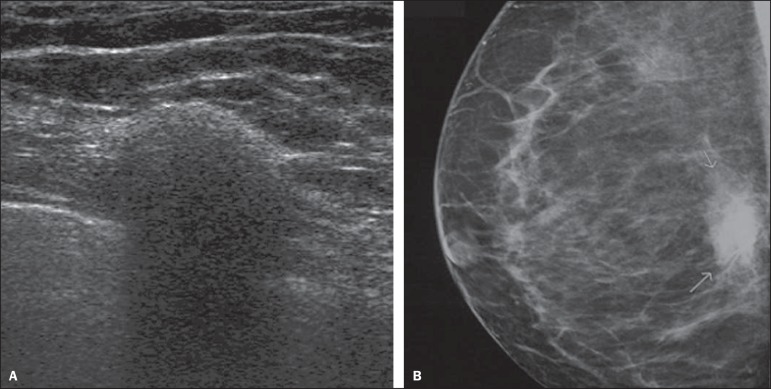
*Steatonecrosis*. On A, ultrasonography showing hyperechoic, ovoid nodule with indistinct margins and posterior acoustic shadowing. On B, correlation with mammographic study - focal asymmetry is observed in the posterior third of the breast.

It is a common entity that may result from trauma, but in most cases it occurs after surgery or radiotherapy. Its appearance depends upon the presence of histiocytic infiltrate, hemorrhage, fibrosis or calcification^(4^. At US it presents with varied aspects, as a focal hyperechoic subcutaneous area, anechoic mass with posterior acoustic shadowing, solid or cystic mass with internal echoes, or a cystic mass with mural nodules (Figure 3)^(4)^.

### Fibroadenoma

**Figure 4:**
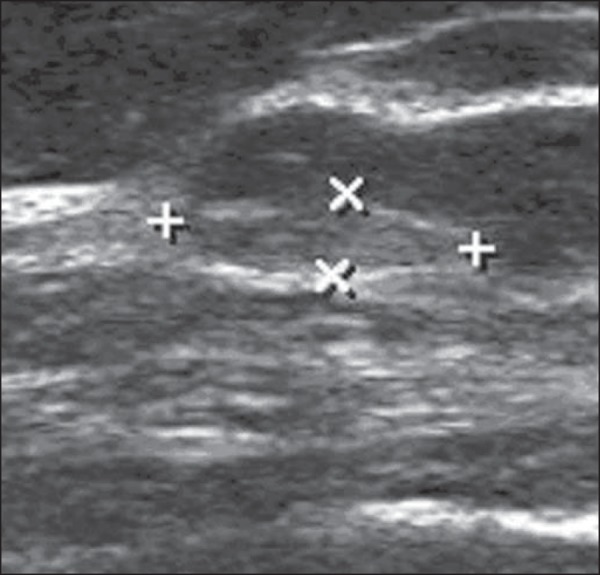
*Fibroadenoma*. Follow-up ultrasonography showing hyperechoic, ovoid nodule with circumscribed margins and largest axis parallel to the skin.

Fibroadenoma is the third most common cause of biopsy in cases of benign breast conditions. The maximum incidence occurs at the third decade of life, with a second peak at the fifth decade. At US, it presents with an elliptical or slightly lobulated shape, and the axis with orientation parallel to the skin, isoechoic or slightly hypoechoic echogenicity, a fine, mobile and slightly compressible echogenic capsule. In 3.1% of cases, fibroadenomas are remarkably hypoechoic, and in 0.9%, either completely or partially hyperechoic (Figure 4). This is due to the presence of either smaller or greater proportions of epithelial and stromal elements^(9)^. As it degenerates, internal, gross (popcorn) or peripheral (halo sign) calcifications are observed^(8,9)^.

### Phyllodes tumor

**Figure 5:**
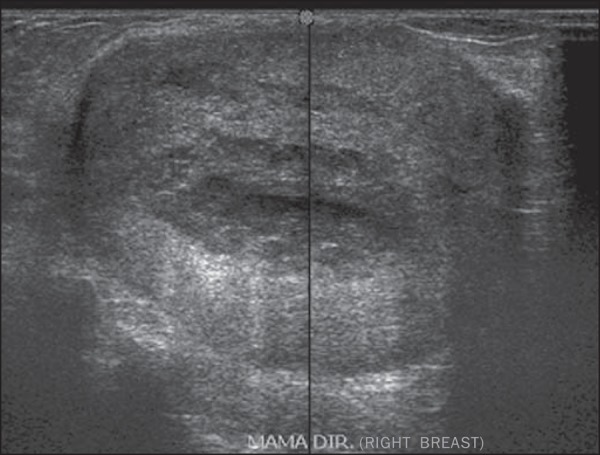
*Malignant phyllodes tumor*. Ultrasonography showing ovoid, echogenic mass with circumscribed and heterogeneous margins, with a central cystic component and posterior acoustic enhancement.

It is responsible for 0.3-1.0% of breast tumors, affecting women aged between 35 and 55 years, as a fast-growing, palpable mass. At US it presents as a hypoechoic and less frequently hyperechoic, solid, well delimited lesion with lobulated margins, occasionally with cystic components, and related to the degree of necrosis and fibrosis (Figure 5)^(8,10)^.

### Hemangioma

**Figure 6:**
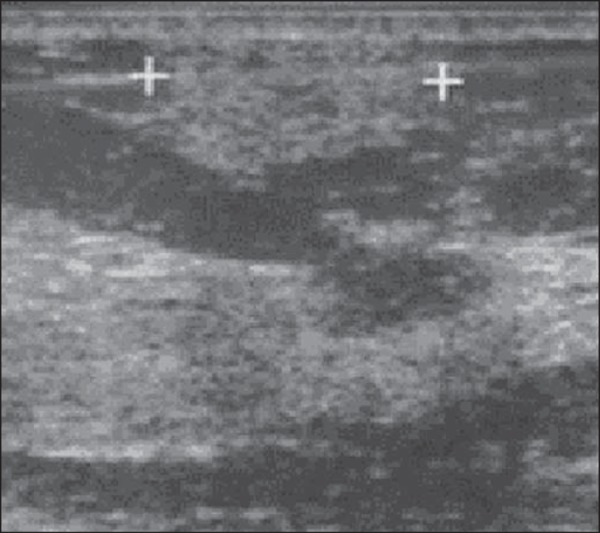
*Hemangioma*. Ultrasonography showing subcutaneous, palpable, hyperechoic nodule with microlobulated contours.

A superficial vascular lesion located in the dermis or in the subcutaneous layer, rarely affecting the breast, with higher incidence in middle-aged women. At US, hemangiomas present with a lobulated or ovoid shape, with well circumscribed margins. Most hemangiomas are either hypoechoic or isoechoic, and may be complex. However, in 33% of cases, they appear as hypoechoic lesions with distal attenuation (Figure 6)^(11)^.

### Intraductal papilloma

**Figure 7:**
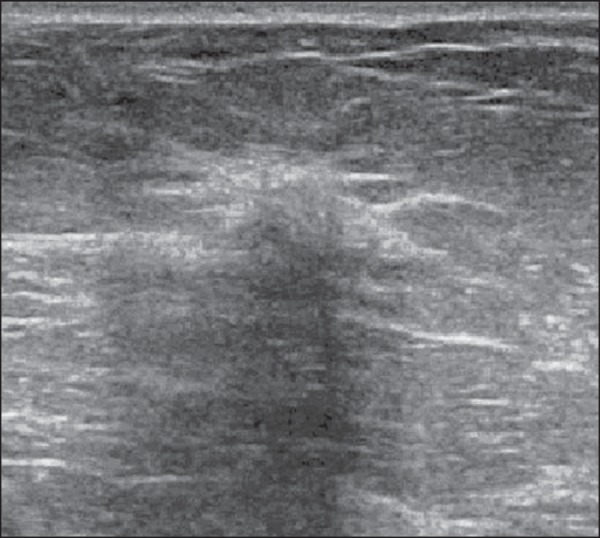
*Intraductal papiloma*. Ultrasonography showing irregular nodule with orientation perpendicular to the skin, and posterior acoustic shadowing.

Intraductal papillomas are polypoid lesions within the breast duct. Generally, they affect perimenopausal women, and the most frequent symptoms are sanguinolent, serous or serosanguinolent discharge. At US, intraductal papillomas are seen as a hypoechoic, solid, round or lobulated nodule, but its echogenicity may be variable. In cases of ductal obstruction, the papilloma may be surrounded by fluid, mimicking a mural nodule within a cyst (Figure 7)^(8,12)^.

### Myofibroblastoma

**Figure 8:**
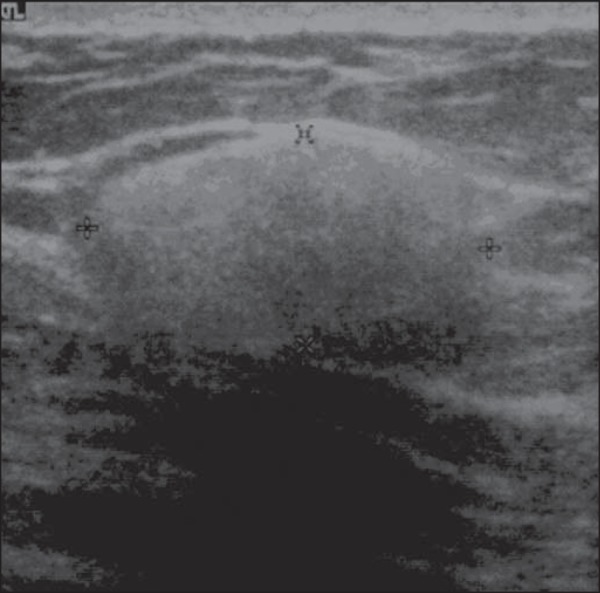
*Myofibroblastoma*. Ultrasonography showing ovoid, hyperechoic nodule with indistinct margins and posterior acoustic shadowing.

It is a rare benign, mesenchymal, spindle cell tumor with varied histological aspect and cellularity, representing differential diagnosis of sarcomatous tumor. This tumor is predominantly reported in men as a circumscribed nodule, generally smaller than 3 cm^(11)^. The radiological characteristics are variable, and, at US. It presents as a solid, well delimited mass that may be hypoechoic, isoechogenic or hyperechoic, depending on the fatty component (Figure 8)^(13)^.

### Pseudoangiomatous stromal hyperplasia

**Figure 9:**
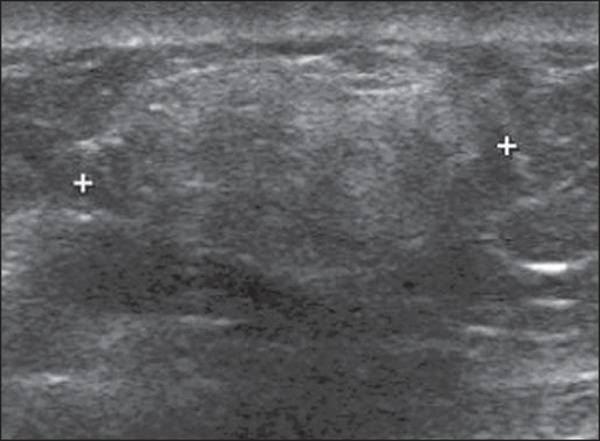
*Pseudoangiomatous stromal hyperplasia*. Ultrasonography demonstrating heterogeneous, predominantly hyperechogenic, ovoid nodule with indistinct posterior margin and subtle posterior acoustic shadowing.

It is a mesenchymal tumor commonly found in perimenopausal women or those undergoing hormone replacement therapy, representing 0.4% of breast lesions. Clinically, it may present as either a palpable nodule or as a diffuse involvement of the breast. At US, they appear as ovoid, heterogeneous and occasionally hyperechogenic lesions (Figure 9)^(5,11)^.

## MALIGNANT LESIONS

### Ductal carcinoma in situ

**Figure 10:**
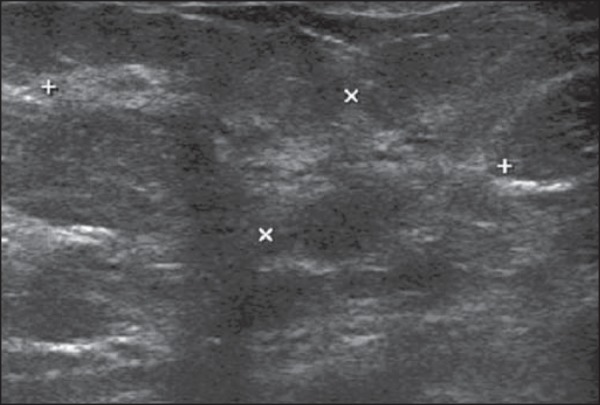
*Ductal carcinoma in situ* in a patient with Paget disease. Ultrasonography showing heterogeneous, irregular area with subtle parenchymal disorganization associated with intermingled microcysts.

The hyperechogenic presentation at US is uncommon, and is reported in less than 0.8% of cases. Certain histological patterns, such as cribriform carcinoma and solid subtypes, together with tumor heterogeneity, are associated with the lesion hyperechogenicity^(13)^. Thus, despite the high negative predictive value for malignancy of hyperechogenic nodules, the lesions should be carefully evaluated as regards shape, margin and hypervascularization, indicating histological evaluation in the presence of suspicious findings (Figure 10)^(13)^.

### Lymphoma

**Figure 11:**
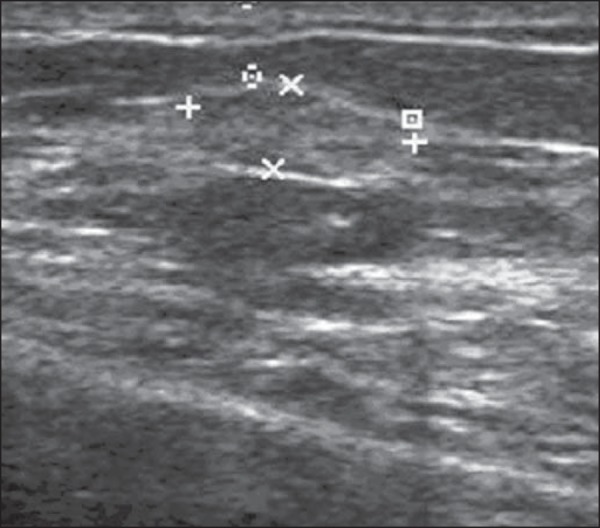
*Lymphoma*. Ultrasonography showing a slightly heterogeneous, regular nodule parallel to the skin. Lesion identified at follow-up of a patient undergoing treatment for non-Hodgkin lymphoma.

It corresponds to 0.1-0.5% of malignant breast lesions. Clinically, it may manifest as a palpable mass. At US, it presents as a hypoechogenic mass with well defined or irregular margins, but the pattern may be heterogeneous with a hyperechogenic halo (Figure 11)^(12)^.

### Invasive ductal carcinoma

**Figure 12:**
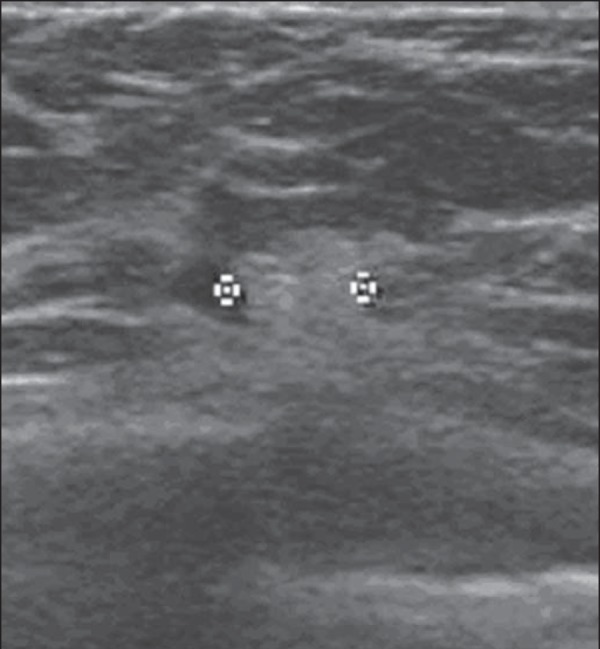
*Invasive ductal carcinoma*. Ultrasonography showing hyperechoic, rounded nodule with indistinct posterior margin.

It represents 75% of invasive breast tumors. At US it presents as a hypoechogenic image with non-circumscribed margins, and in 2% of cases it may be hyperechogenic. Probably, the hyperechogenicity is due to reflective interfaces caused by growth and infiltration of the cellular component, and fatty inclusions involving a poorly perceptible hypoechogenic, hyalinized center (Figure 12)^(4,14)^.

### Metastases

**Figure 13:**
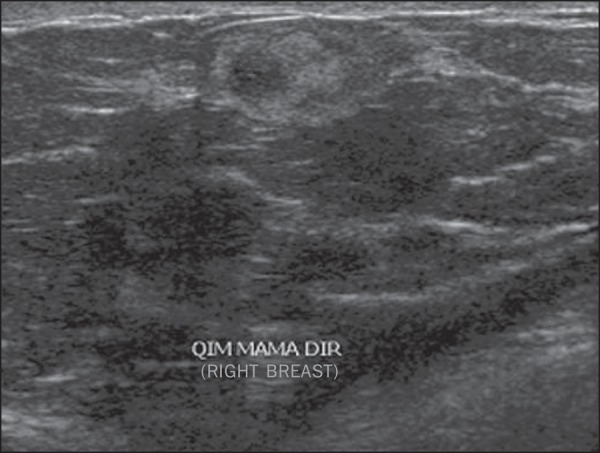
*Metastasis from leiomyosarcoma*. Ultrasonography identifying heterogeneous, palpable nodule with microlobulated margins.

Metastases represent 0.5-2% of malignant breast nodules. The most common primary tumors are lymphoma, melanoma and rhabdomyosarcoma. At US, they present as bilateral, fast-growing, palpable, painless, hypoechogenic nodules with irregular margins (Figure 13)^(11,15)^.

## CONCLUSION

Hyperechogenic breast nodules are uncommon and, despite the high predictive value for benignity, all the sonographic characteristics should be taken into consideration^(16)^. A full appreciation of the most suspicious imaging findings such as non-circumscribed margins and posterior acoustic shadowing, together with appropriate mammographic correlation and clinical context, contribute to determine the most appropriate clinical/surgical approach^(17)^.
